# Graphene-Modulated Terahertz Metasurfaces for Selective and Active Control of Dual-Band Electromagnetic Induced Reflection (EIR) Windows

**DOI:** 10.3390/nano11092420

**Published:** 2021-09-17

**Authors:** Xunjun He, Chenguang Sun, Yue Wang, Guangjun Lu, Jiuxing Jiang, Yuqiang Yang, Yachen Gao

**Affiliations:** 1 Key Laboratory of Engineering Dielectric and Applications (Ministry of Education), Harbin University of Science and Technology, Harbin 150080, China; scg0121@foxmail.com (C.S.); yuewang@163.com (Y.W.); jiangjiuxing@hrbust.edu.cn (J.J.); 2 College of Electronic Engineering, Guangxi Normal University, Guilin 541004, China; lv-guangjun@163.com; 3 School of Electronic and Information Engineering, Guangdong Ocean University, Zhanjiang 524088, China; 4 College of Electronic Engineering, Heilongjiang University, Harbin 150080, China

**Keywords:** graphene-functionalized metasurfaces, dual-reflection window, selective control, electric doping

## Abstract

Currently, metasurfaces (MSs) integrating with different active materials have been widely explored to actively manipulate the resonance intensity of multi-band electromagnetic induced transparency (EIT) windows. Unfortunately, these hybrid MSs can only realize the global control of multi-EIT windows rather than selective control. Here, a graphene-functionalized complementary terahertz MS, composed of a dipole slot and two graphene-integrated quadrupole slots with different sizes, is proposed to execute selective and active control of dual-band electromagnetic induced reflection (EIR) windows. In this structure, dual-band EIR windows arise from the destructive interference caused by the near field coupling between the bright dipole slot and dark quadrupole slot. By embedding graphene ribbons beneath two quadrupole slots, the resonance intensity of two windows can be selectively and actively modulated by adjusting Fermi energy of the corresponding graphene ribbons via electrostatic doping. The theoretical model and field distributions demonstrate that the active tuning behavior can be ascribed to the change in the damper factor of the corresponding dark mode. In addition, the active control of the group delay is further investigated to develop compact slow light devices. Therefore, the selective and active control scheme introduced here can offer new opportunities and platforms for designing multifunctional terahertz devices.

## 1. Introduction

Over the last few decades, the electromagnetic induced transparency (EIT) first observed in the atomic system has aroused a lot of interest because it allows the enhancement of transmission within a narrow frequency range along with strong dispersion properties and displays promising engineering prospects [1,2,3]. However, its implementation requires rigorous experimental conditions, severely hindering practical applications [4]. With the rapid development of metasurfaces (MSs), recently, the MS-based EIT-like effect has attracted tremendous attention owing to flexible design and easy fabrication [5]. Currently, the EIT-like effect in terahertz (THz) MSs has been widely investigated and experimentally demonstrated by bright–dark mode coupling [6,7] or bright–bright mode coupling [8,9]. Unfortunately, most of these structures can only realize a single transparency window, in which the resonance strength of the window is fixed after fabrication, restricting their application fields [10].

To overcome the above limitation, a variety of THz MSs with multi-band EIT windows were designed to actively modulate resonance properties by means of different approaches, including mechanical movement, phase change material, and graphene. For example, Devi et al. designed a concentrically coupled asymmetric THz MS to obtain a dual-band EIT effect; moreover, the dual-band EIT effect can be effectively modulated by rotating the element angle or by changing the asymmetric degree of the unit cell [11]. Chen et al. demonstrated an actively tunable dual-band EIT window in a VO_2_-integrated MSs [12]. In addition, the last decade saw the advent of graphene with flexible tunability, which has given rise to unprecedented progress in actively tunable microwave [13,14], terahertz [15,16], and optical [17] devices. According to similar principles, Gao et al. reported an actively tunable dual plasmon-induced transparency (PIT) MS based on graphene-patterned structures [18]. Meanwhile, three-band tunable EIT MSs (or those with more than three bands) were also investigated by stacking multilayer graphene/dielectric structures [19,20]. Among all of the previously mentioned MS structures, they primarily rely on uniform excitation to implement the global control of multi-band windows rather than selective control; that is, each one of the multi-band windows cannot be independently controlled or modulated. With the increasing demand for selective and tunable control systems, however, simple and multifunctional MSs are highly desirable for reconfigurable THz devices in wireless communications and data storage systems [21,22,23].

Here, we propose a scheme to obtain a dual-band EIR window in a graphene-functionalized complementary THz MS consisting of two dolmen-like slot structures with different sizes and two graphene ribbons connecting to different top gates. The basic article structure is as follows: First, the structural design and parameter selection of the proposed MS are described in detail, as well as the simulation setting. Second, the formation process and the physical mechanism of two EIR windows are elucidated through the analysis of different element arrays in a unit cell and field distributions. Then, the selective and active control of two EIR windows are further investigated by tailoring the Fermi energy of the corresponding graphene ribbons via electrostatic doping. Moreover, the field distributions and classic three-oscillator model are employed to discover the selective and active control mechanism of two EIR windows. Finally, the active control of the group delay originating from strong phase dispersion is also investigated to develop compact slow light devices. Therefore, the results obtained for our devices could provide new opportunities and platforms for the design and development of multi-function terahertz devices.

## 2. Structures, Materials and Methods

**Figure 1:**
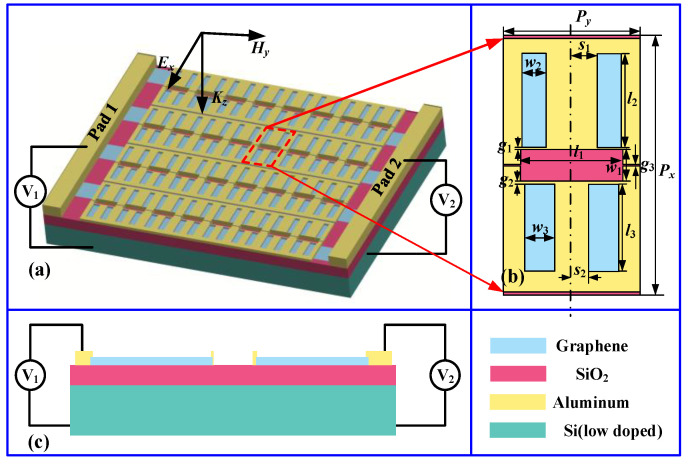
The proposed graphene-functionalized EIR metasurface: (**a**) three-dimensional schematic of the EIR metasurface, (**b**) schematic of the unit cell; the geometric parameters are as follows: *l*_1_ = 120 μm, *l*_2_ = 109 μm, *l*_3_ = 100 μm, *w*_1_ = 36 μm, *w*_2_ = 28 μm, *w*_3_ = 35 μm, *s*_1_ = 30 μm, *s*_2_ = 20 μm, *g*_1_ = 2 μm, *g*_2_ = 3.4 μm, *g*_3_ = 2 μm, *P_x_* = 30 μm and *P_y_* = 160 μm, and (**c**) cross-sectional view of unit cell.

Figure 1 shows the schematic of the proposed graphene-functionalized complementary THz MS structure, in which the metal Al film, graphene layer, dielectric layer, and substrate are represented by yellow, blue, red, and green, respectively. The unit cell is composed of the metallic slot structures and two patterned monolayer graphene ribbons, and the metallic slot structures comprise a dipole slot serving as a bright resonator and two quadrupole slots of different sizes serving as two different dark resonators fabricated by etching the Al film, thus forming two dolmen-like slot structures with a communal dipole slot, as shown in Figure 1a,b. When the designed structure is excited by the x-polarized incident wave, two distinct EIR windows would be induced by tuning the near field coupling between the corresponding bright-dark modes. To realize selective control of the two EIR windows, two quadrupole slots are mutually separated by a small gap in the center of the dipole slot and are deposited on the graphene ribbons connected by different top gates, as shown in Figure 1c. Compared to traditional structures, the key feature of our structure is that it can realize the selective and active control of the two windows by adjusting the Fermi energy of the corresponding graphene ribbon via electric doping.

To investigate the THz response of the proposed complementary MS, numerical calculations were conducted using the FDTD method, in which the boundary conditions in the x- and y-directions were set as the unit cell, and the z-direction was left open. The x-polarized incident light was normally illuminated on the surface of the designed complementary MS along the z-direction, as displayed in Figure 1a. Moreover, the size and number of the rational mesh were used to meet the accuracy requirements. In addition, the lossy metal Al with the DC conductivity of 3.56 × 10^7^ S/m was employed as the slot resonators during the calculation [24], while the low-doped silicon with a permittivity of 11.7 and thickness of 300 μm and a SiO_2_ film with a permittivity of 3.9 and thickness of 30 μm were used as the substrate and dielectric layer, respectively. Since the graphene is composed of a one atom-thick layer of carbon atoms arranged in a hexagonal pattern, generally, the graphene is assumed to be a 1.0-nm-thick homogenous layer in order to facilitate simulation [25], while the effective permittivity is written as [26]:

(1) $$\varepsilon_{g}=1+j\frac{\sigma_{g}}{\omega \varepsilon_{0}t_{g}}$$

Here, *ω* is the angular frequency, *t*_g_ is the single-layer graphene thickness, and *ε*_0_ is the vacuum permittivity. In the terahertz range, the graphene conductivity *σ*_g_ is described by the following expression [27]:

(2) $$\sigma =-j\frac{e^{2}k_{B}T}{\pi \hbar^{2}(\omega -j2\tau^{-1})}(\frac{E_{F}}{k_{B}T}+2\ln(e^{-E_{F}/k_{B}T}+1))$$

where *e* is the electron charge, ℏ=h/2π is the reduced Planck’s constant, *k_B_* is the Boltzmann’s constant, *T* is the Kelvin temperature, and *E_F_* is the Fermi energy of graphene. *τ* is the carrier relaxation time described by $\tau =\left(\mu E_{F}\right)/\left(ev_{F}^{2}\right)$; here, *T* = 300 K, $\mu =3000\;{\mathrm{cm}}^{2}/V\cdot s$, and $v_{F}=1.1\times 10^{6}\;m/s$ are used in our calculations [28]. According to Equation (2), the conductivity of graphene can be actively tuned by shifting the Fermi energy of graphene, while the Fermi energy of the graphene can be dynamically controlled by the voltages of the top gates (as shown in Figure 1c); that is, the conductivity of the single-layer graphene can be actively modulated by the top-gate voltages, which is different from the electro-intercalation way applied in multilayer graphenes [29], and the corresponding expression is as follows [30]:

(3) $$E_{F}=\hbar v_{F}\sqrt{\pi \varepsilon_{0}\varepsilon_{r}V_{g}/ed_{s}}$$

Here, *ε*_r_ and *d*_s_ are the relative permittivity and thickness of the SiO_2_ dielectric layer. Thus, by tuning the conductivity of graphene through the gate voltage *V_g_*, the THz properties of the designed device could be effectively controlled.

## 3. Evolution Mechanism of Two EIR Windows

**Figure 2:**
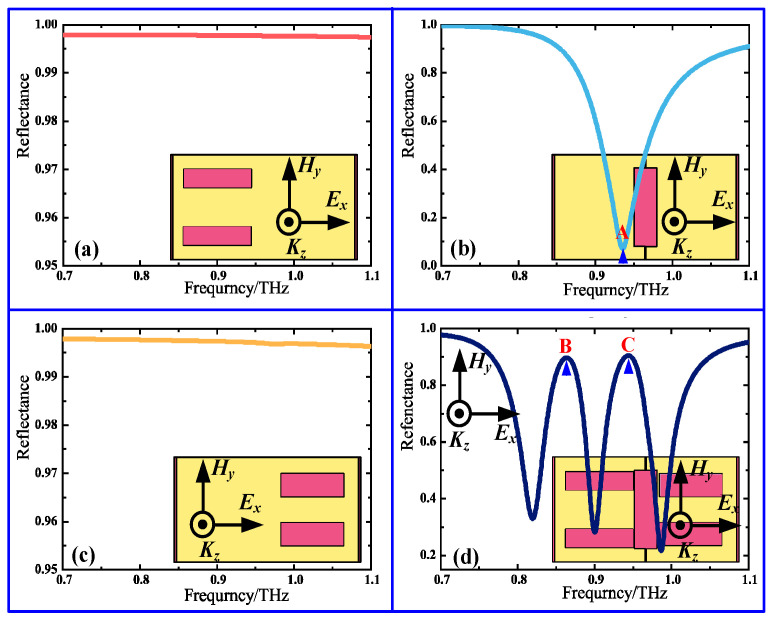
Reflection spectra of four different complementary MSs for *x*-polarized incident waves: (**a**) longer quadrupole slot array alone, (**b**) dipole slot array alone, (**c**) shorter quadrupole slot array alone, and (**d**) their combined structure.

To disclose the formation process of dual-band EIR effect, first, the reflection spectra of four different complementary MSs, consisting of a longer quadrupole slot array alone, a dipole slot array alone, and shorter quadrupole slot array alone as well as their combined structure were calculated for the *x*-polarized incident wave, as presented in Figure 2. For the individual dipole slot, a sharp reflection dip is observed at 0.93 THz due to direct coupling with the *x*-polarized incident wave, which serves as a bright mode, as shown by the shallow blue line in Figure 2b. For the individual longer or shorter quadrupole slots, by contrast, the reflection amplitude is almost close to 1.0, and no resonance appears in the interesting frequency range due to the structural symmetry, as displayed by the red line in Figure 2a or the orange line in Figure 2c. Thus, the quadrupole slot acts as a dark mode. When three slot elements are combined together to form the unit cell shown in Figure 1b, two distinct EIR windows with a resonance strength of more than 90% in the reflection spectrum appear at 0.86 THz and 0.94 THz due to the destructive interference caused by the near field coupling between the corresponding bright–dark mode, as represented by the deep blue line in Figure 2d. 

**Figure 3:**
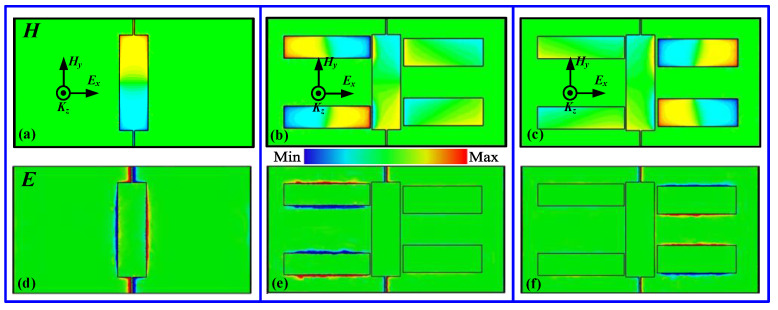
Magnetic and electric field distributions at different resonances: (**a**,**d**) at marked point “A” in Figure 2b, (**b**,**e**) at marked point “B” in Figure 2d, (**c**,**f**) at marked point “C” in Figure 2d.

To further understand the physical mechanism of the two EIR windows, the field distributions of the designed structure were calculated when the incident wave was polarized along the *x*-direction, and the corresponding field distributions at three different resonance frequencies, marked as the symbols “A”, “B”, and “C” in Figure 2b,d, are displayed in Figure 3. For the dipole slot alone in Figure 3a,d, the magnetic fields are strongly focused on both ends of the dipole slot, while the electric fields are concentrated in the center, indicating the excitation of a dipole resonance, as presented in Figure 2b. For the combined structure, the magnetic and electric fields at two resonance peaks are confined to both ends and the center of the corresponding quadrupole slot in which the fields are opposite to each other, while that of the dipole slot is fully restrained, as shown in Figure 3b–f. Such field distributions imply that there is a notable energy shift between the dipole slot and the quadrupole slot due to strong near field coupling; as a result, the two pronounced EIR windows are excited by the destructive interference caused by the near field coupling, as shown in Figure 2d.

## 4. Selective and Active Modulation of Two EIR Windows

**Figure 4:**
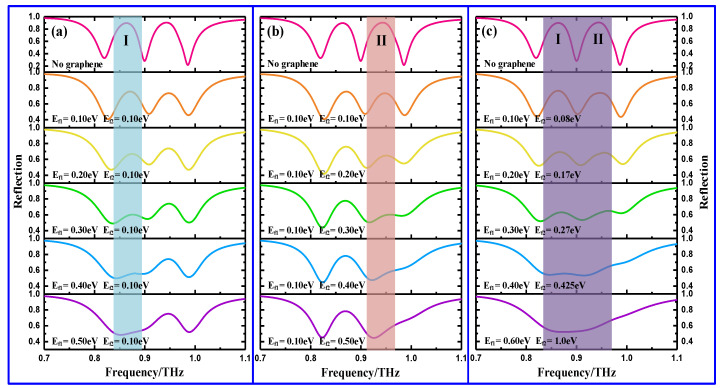
Simulated reflectance spectra for three different modulation ways: (**a**) shifting E_f1_ only, (**b**) shifting E_f2_ only, and (**c**) simultaneously shifting E_f1_ and E_f2_.

To examine the selective and active modulation of the two windows, we next investigated the tunable characteristics of the graphene-functionalized MS by tailoring the Fermi energies of two graphene ribbons, in which the Fermi energy of the graphene ribbons beneath the long and short quadrupole slots are denoted by E_f1_ andE_f2_, respectively. Thus, by independently tuning any of E_f1_ and E_f2_ or by synchronously tuning E_f1_ and E_f2_, the graphene-functionalized MS can execute three different ways to control the resonance intensity of the two windows, as shown in Figure 4. As shown in the first row of Figure 4, two distinct EIR windows are observed at 0.86 THz and 0.94 THz without graphene. To facilitate expression, the low- and high-frequency windows are, described as Window I and Window II in the following. Once the graphene ribbons have been embedded in the complementary structure, however, it is noted that the profile features of two windows remain still similar to that of the one no graphene ribbon, with the exception of a slight decline in the strength (not shown here). The slight decrease in strength can be ascribed to the intrinsic conductivity of the graphene, which can shield the near field coupling between the two modes, and similar results have also been observed in previous reports [31]. As the Fermi energy increases in the graphene ribbon, the amplitude of the EIR window should further decline. Figure 4a shows the intensity modulation of Window I when E_f1_ is individually tailored and when E_f2_ is maintained at 0.1 eV. It can be clearly observed that when E_f1_ increases from 0.1 eV to 0.5 eV, the intensity of Window I changes dramatically, while Window II remains unchanged. For example, as E_f1_ increases from 0.1 eV to 0.4 eV, the amplitude of Window I decreases from 76% to 56%. With further increases to 0.5 eV, however, Window I is totally switched off and becomes a broad resonance valley. The extinction of Window I can be attributed to the increasing conductivity of the graphene. Thus, Window I can be independently controlled by merely tuning E_f1_. To individually tune E_f2_ from 0.1 eV to 0.5 eV, a similar change is also observed in Window II, with dependent control of Window II being obtained by Window I shifting E_f2_ on its own, as shown in Figure 4b. The intensity change of the two windows that occurs when simultaneously tuning E_f1_ and E_f2_, however, is shown in Figure 4c. It can be seen that as E_f1_ and E_f2_ increase, the two windows weaken gradually and are annihilated, eventually becoming a considerable broad resonance dip, different from that of Figure 2b. As a result, the two windows can also realize an on-to-off switch by synchronously shifting E_f1_ and E_f2_. In addition, it is noteworthy that during the entire modulation process, the locations of the two windows remain almost unchanged. To evaluate the modulation effect of the three control methods discussed above, the intensity modulation depth of the two windows is calculated by Δ*R*/*R*_0_ = (*R*_0_ − *R*_g_)/*R*_0_, where *R*_0_ and *R*_g_ are the reflectance amplitude of the window without and with graphene, respectively. Thus, the corresponding intensity modulation depths are 46% when shifting E_f1_ alone, 46% when shifting E_f2_ alone, and more than 40% when simultaneously shifting E_f1_ and E_f2_. Therefore, the two windows can be selectively and actively modulated by tuning the Fermi energy of the corresponding graphene ribbons. Based on this scheme, a MSs with more windows can also be realized, in which each EIR window can be independently and actively controlled.

**Figure 5:**
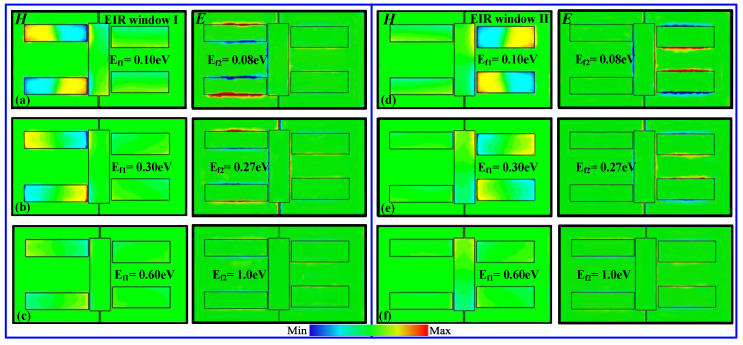
Magnetic and electric field distributions of two EIR windows at different Fermi energies: (**a**) E_f1_ = 0.10 eV and E_f2_ = 0.08 eV, (**b**) E_f1_ = 0.30 eV and E_f2_ = 0.27 eV, and (**c**) E_f1_ = 0.60 eV and E_f2_ = 1.0 eV for EIR Window I and (**d**) E_f1_ = 0.10 eV and E_f2_ = 0.08 eV, (**e**) E_f1_ = 0.30 eV and E_f2_ = 0.27 eV, and (**f**) E_f1_ = 0.60 eV and E_f2_ = 1.0 eV for EIR Window II.

To further demonstrate the claims that the above-mentioned two windows can be selectively and actively controlled by shifting the Fermi energy of the corresponding graphene ribbons, the field distributions at two window frequencies are calculated at different Fermi energies, as shown in Figure 5. Figure 5 presents the magnetic and electric field distributions for different E_f1_ and E_f2_ values obtained by simultaneously doping two graphene ribbons. At E_f1_ = 0.10 eV and E_f2_ = 0.08 eV, the graphene ribbon can be regarded as a semiconductor with low conductivity, and the confined magnetic and electric fields are strongly focused on the corresponding quadrupole slots (see Figure 5a,d), indicating the occurrence of two windows. However, when E_f1_ and E_f2_ are gradually increased up to 0.6 eV and 1.0 eV, respectively, the confined fields in the two quadrupole slots initially exhibit considerable attenuation and, ultimately, total annihilation; as a result, this leads to the two windows switching off, as shown in Figure 5b,c,e,f. The change in the magnetic and electric field strengths can be attributed to the increased conductivity of the graphene ribbon, which is able to shield the near field coupling between the bright and dark modes. Here, and in the following section, only the synchronous doping method for the two graphene ribbons is analyzed due to the similar mechanism, while other methods (selective doping) are not further discussed. In addition, it is noted that during the entire tuning process, the confined fields in the dipole slots are also suppressed due to the fact that it increasingly dampens itself, as demonstrated in the last row of Figure 4.

**Figure 6:**
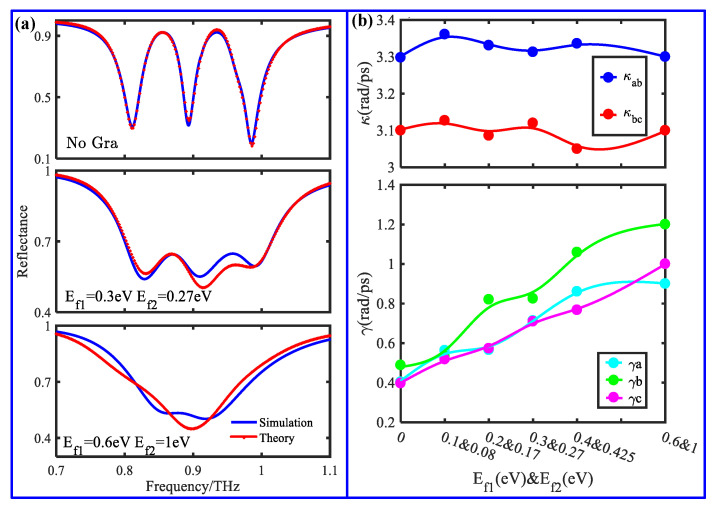
(**a**) Comparison of simulated and theoretical results for different Fermi energies and (**b**) fitting parameters *κ_ab_*, *κ_bc_*, γ*_a_*, γ*_b_*, and γ*_c_* as the function of the Fermi energy.

To discover tunable mechanism, a classic three-oscillator coupled model was employed to describe the designed graphene-functionalized MS composed of a dipole slot and two graphene-integrated quadrupole slots. In this model, the dipole slot is denoted by the oscillator *b* being excited directly by the incident wave *E*, and the two quadrupole slots are denoted by the oscillators *a* and *c*, which are being excited indirectly by near field coupling with oscillator *b*. Thus, the coupled differential equations among the three oscillators are expressed as follows [12]:

(4) $${\ddot{x}}_{a}(t)+\gamma_{a}{\dot{x}}_{a}(t)+\omega_{a}x_{a}(t)+\kappa_{ab}^{2}x_{b}(t)=0$$

(5) $${\ddot{x}}_{b}(t)+\gamma_{b}{\dot{x}}_{b}(t)+\omega_{b}x_{b}(t)+\kappa_{ab}^{2}x_{a}(t)+\kappa_{bc}^{2}x_{c}(t)=qE$$

(6) $${\ddot{x}}_{c}(t)+\gamma_{c}{\dot{x}}_{c}(t)+\omega_{c}x_{c}(t)+\kappa_{bc}^{2}x_{b}(t)=0$$

where (*x_a_*, *γ_a_*), (*x_b_*, *γ_b_*), and (*x_c_*, *γ_c_*) are the resonance intensities and damping factors of three oscillators *a*, *b*, and *c*, respectively. *ω_a_*, *ω_b_*, and *ω_c_* are the resonance frequencies of the three resonators before coupling. *κ_ab_* denotes the coupling coefficient between resonator *b* and oscillator *a*, while *κ_bc_* is the coupling coefficient between resonator *b* and oscillator *c*. q represents the coupling coefficient between resonator *b* and the incident wave. After solving Equations (4)–(6), the energy dissipation of the graphene-functionalized complementary MS as the functions of the frequency is given as follows:

(7) $$P(\omega )\propto \frac{C_{a}C_{c}}{C_{c}\kappa_{ab}^{2}+C_{a}\kappa_{bc}^{2}-C_{a}C_{b}C_{c}}$$

where *C_j_ = ω_j_*^2^ + *iωγ_j_* − *ω*^2^ (*j* = *a*, *b*, *c*). According to Equation (7), the simulated reflection spectrum can be theoretically fitted, and the corresponding fitting results are shown in Figure 6. It can be seen from Figure 6a that for synchronous doping, the theoretical fitting results show reasonable agreement with the simulated results. Moreover, based on a similar fitting method, the fitting and simulated results can also exhibit very excellent agreement for selective doping (not shown here).

To quantitatively explain the active modulation of the two windows in the synchronous doping situation shown in Figure 4c, the fitting parameters *κ_ab_, κ_bc_, γ_a_, γ_b,_* and *γ_c_* as the functions of E_f1_ and E_f2_ are extracted during the fitting calculations and are represented in Figure 6b. During synchronous doping, the coupling coefficients *κ_ab_* and *κ_bc_* stay nearly constant with E_f1_ and E_f2_, while the amplitudes of the damping factors *γ_a_* and *γ_c_* exhibit a linear increase. When E_f1_ and E_f2_ are increased up to 0.6 eV and 1.0 eV, respectively, *γ_a_* and *γ_c_* become large enough to suppress the excitation of two dark elements, as a result, whichleads to the complete disappearance of the two windows. In addition, it is observed that the damping factor *γ_a_* arises gradually with the increase of E_f1_ and E_f2_, which is consistent with the field distributions shown in Figure 5. Therefore, the active modulation of the reflection window can be attributed to the increased damping factor of the dark element arising from the increasing conductivity of the doped graphene ribbon.

## 5. Tunable Slow-Light Applications

**Figure 7:**
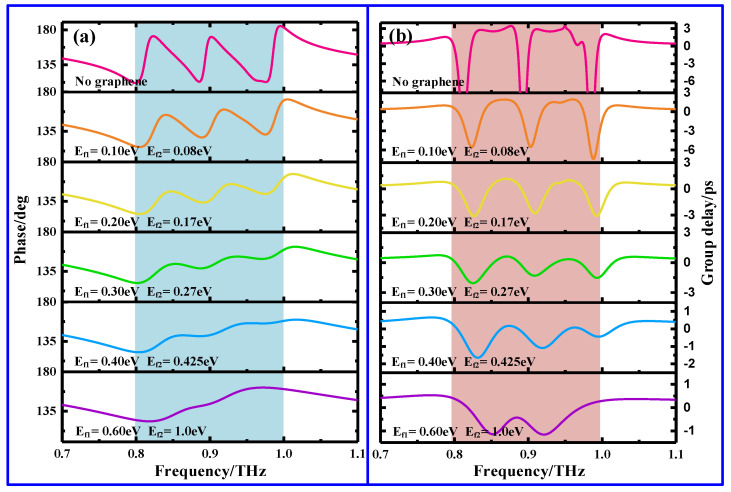
Reflection phase shift (**a**) and group delay (**b**) of the proposed graphene-functionalized MS with different E_f1_ and E_f2_.

As it is widely known, one remarkable feature of the EIT effect is that it is accompanied by strong phase dispersion slowing down the light speed, thus exploiting slow light devices. Generally, slow light effect can be quantitatively characterized by a group delay (*τ*_g_ = −*dφ* /*dω*, in which *φ* and *ω* = 2π*f* are the phase shift and the angular frequency) [32], as shown in Figure 7. Figure 7a shows the reflection phase spectra for different E_f1_ and E_f2_. As expected, there is a notable phase dispersion around two EIR windows; moreover, the dispersion in phase shift progressively weakens with E_f1_ and E_f2_. Figure 7b shows the group delays of the corresponding phases. When there is not graphene ribbon, the group delays near the two EIR windows can be up to 2.6 ps and 3.4 ps, respectively, indicating that light waves propagate through the designed MS sample with a slower group velocity than they do through air. Such a large group delay can be ascribed to the steepest phase shift change within the reflection window. Once the graphene ribbons have been integrated to form a graphene-functionalized MS, however, it can be easily discerned that when E_f1_ and E_f2_ are 0.30 eV and 0.27 eV, respectively, the two group delays are suppressed to 0.36 ps and 0.32 ps, owing to the phase dispersion being weakened. As E_f1_ and E_f2_ are further increased up to 0.60 eV and 1.0 eV, the group delay totally disappears and becomes a negative value. Moreover, the selective control of the two delay groups can be also observed by doping the corresponding graphene ribbon (not shown here). By doping the corresponding graphene ribbon, the graphene-functionalized MS can also achieve the ability to selectively control the group delay, which has potential applications for the development of compact slow light devices.

## 6. Conclusions

In conclusion, we have numerically demonstrated the selective and active control of two EIR windows in a graphene-functionalized complementary MS consisting of a dipole slot and two graphene-integrated quadrupole slots of different sizes. In the structure, two EIR windows are induced by destructive interference originating from near field coupling between the dipole and the quadrupole slots and can be selectively or synchronously modulated by doping the corresponding graphene ribbons. The theoretical model and field distributions discovered that such active control can be ascribed to the increased damping factor of the dark element. In addition, the group delay is also actively controlled by doping the corresponding graphene ribbons, which is well-suited for the development and application of compact slow light devices. Based on this scheme, the selective and active control of multiple windows can further extend to other functionalized MSs by implanting different dark elements integrated with active materials, which offer new opportunities and platforms for the design of multifunctional terahertz devices.

## Data Availability

Data is contained within the article.
